# Biological Control of Aflatoxin in Maize Grown in Serbia

**DOI:** 10.3390/toxins12030162

**Published:** 2020-03-05

**Authors:** Zagorka Savić, Tatjana Dudaš, Marta Loc, Mila Grahovac, Dragana Budakov, Igor Jajić, Saša Krstović, Tijana Barošević, Rudolf Krska, Michael Sulyok, Vera Stojšin, Mladen Petreš, Aleksandra Stankov, Jelena Vukotić, Ferenc Bagi

**Affiliations:** 1 Faculty of Agriculture, University of Novi Sad, 21000 Novi Sad, Serbia; zagorka.savic@polj.uns.ac.rs (Z.S.); marta.loc@polj.uns.ac.rs (M.L.); mila@polj.uns.ac.rs (M.G.); dbudakov@polj.uns.ac.rs (D.B.); igor.jajic@stocarstvo.edu.rs (I.J.); sasa.krstovic@stocarstvo.edu.rs (S.K.); tijana.doroski@gmail.com (T.B.); stojsinv@polj.uns.ac.rs (V.S.); mladen.petres@polj.uns.ac.rs (M.P.); aleksandra.stankov@polj.uns.ac.rs (A.S.); jelena.medic@polj.uns.ac.rs (J.V.); bagifer@polj.uns.ac.rs (F.B.); 2 Institute of Bioanalytics and Agro-Metabolomics, Department IFA-Tulin, University of Natural Resources and Life Sciences Vienna (BOKU), A-3430 Tulln, Austria; rudolf.krska@boku.ac.at (R.K.); michael.sulyok@boku.ac.at (M.S.); 3 Institute for Global Food Security, School of Biological Sciences, Queens University Belfast, University Road, Belfast BT7 1NN, UK

**Keywords:** aflatoxin, *Aspergillus flavus*, biological control, atoxigenic strain, maize, Serbia

## Abstract

*Aspergillus flavus* is the main producer of aflatoxin B1, one of the most toxic contaminants of food and feed. With global warming, climate conditions have become favourable for aflatoxin contamination of agricultural products in several European countries, including Serbia. The infection of maize with *A. flavus*, and aflatoxin synthesis can be controlled and reduced by application of a biocontrol product based on non-toxigenic strains of *A. flavus*. Biological control relies on competition between atoxigenic and toxigenic strains. This is the most commonly used biological control mechanism of aflatoxin contamination in maize in countries where aflatoxins pose a significant threat. Mytoolbox Af01, a native atoxigenic *A. flavus* strain, was obtained from maize grown in Serbia and used to produce a biocontrol product that was applied in irrigated and non-irrigated Serbian fields during 2016 and 2017. The application of this biocontrol product reduced aflatoxin levels in maize kernels (51–83%). The biocontrol treatment had a highly significant effect of reducing total aflatoxin contamination by 73%. This study showed that aflatoxin contamination control in Serbian maize can be achieved through biological control methods using atoxigenic *A. flavus* strains.

## 1. Introduction

Aflatoxins are the most common contaminants of important agricultural commodities including maize, cottonseed, peanuts, and pistachio nuts. *Aspergillus flavus* and related species produce aflatoxins, which are secondary metabolites that can adversely affect human health and food security in warm agricultural areas [1,2]. Aflatoxins are potent, naturally-occurring carcinogens that can suppress the immune system and induce hepatocellular carcinoma, which can cause mortality in humans and livestock [3,4]. Aflatoxin B1 (AFB1), which is classified as a Group 1a carcinogen by the International Agency for Research on Cancer [5], is the most common and toxic of the four major aflatoxins B1, B2, G1, and G2. Aflatoxin concentration in food and feed is strictly regulated by international organisations due to the significant impacts on human health [6]. Products contaminated by aflatoxins have limited value and access to markets, resulting in significant economic losses [3].

Environmental and biological factors, such as increased temperatures, droughts, pest damages, host susceptibility to infection, and the aflatoxin-producing potential of fungi, have combined to raise aflatoxin contamination levels above regulated limits [7,8]. Aflatoxin contamination can occur after crop maturity when the crops are exposed to high temperature and humidity levels, which are conducive to fungal infection and may result in an increase of aflatoxin accumulation [9]. Therefore, aflatoxin contamination can start and continue after cropping, but also during storage, transport, processing, and handling [10].

In Serbia during the summer of 2012 the appearance of *A. flavus* in maize was a result of extremely stressful environmental conditions that included high air temperatures and little precipitation, and was reported as an emerging food and feed safety threat. Natural occurrences of aflatoxins are uncommon under Serbia’s typical climatic conditions; however, because mycotoxin occurrence is climate-dependent [11], recent climate changes have become significant causal agents of food and feeds safety issues in Serbia [12,13].

Aflatoxins have received considerable research due to global consumer concerns related to the presence of aflatoxins in the food supply. Understanding the biology, epidemiology, and occurrence of aflatoxin-producing fungi and the development of advanced technologies for reducing these fungi are urgently needed. Biological control methods based on the competitive exclusion of toxigenic strains by atoxigenic strains have been developed as an innovative strategy for reducing aflatoxin accumulation. Atoxigenic strains displace aflatoxin producers by competing with other toxigenic and atoxigenic strains for infection sites and essential nutrients during crop development. This competition has resulted in significant reductions in aflatoxin contamination in harvested grains [14,15,16,17].

Researching the genetic diversity within aflatoxin-producing fungi population in Serbia is highly important for determining the etiology of aflatoxin contamination and for optimising the selection of native atoxigenic *A. flavus* genotypes that can be used for aflatoxin biological control products targeted to local agroecosystems. The diverse environmental conditions and soil microbiomes that are found in different locations will favour the use of native *A. flavus* strains for local agroecosystems. Thus, native *A. flavus* strains are expected to provide better results than introduced exotic genotypes [18]. This paper highlights recent research in Serbia that was designed to improve the management of aflatoxin contamination of maize using native atoxigenic isolates. Isolates of selected native atoxigenic genotypes were applied to maize crops prior to flowering as a biological control method with the goal of reducing aflatoxin contamination.

## 2. Results

### 2.1. Monitoring Deletions in the Aflatoxin Biosynthesis Isolate Mytoolbox Af01 Gene Cluster

**Figure 1:**
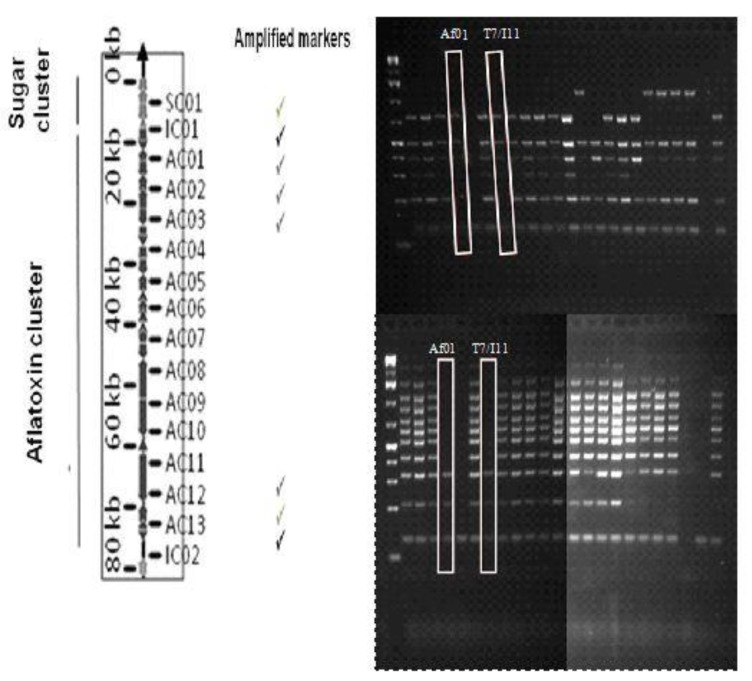
Images of multiplex PCR products aligned to a schematic diagram from Callicot and Cotty [19] of chromosome 3 containing the aflatoxin cluster.

**Figure 2:**
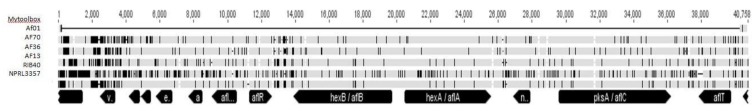
Comparative view of the missing 40 kb region in atoxigenic strain (Mytoolbox Af01), toxigenic *A. flavus* strains (AF70, AF13, NPRL3357), atoxigenic *A. flavus* strain (AF36), and atoxigenic *A. oryzae* strain (RIB40).

Twenty fungal isolates determined to be *A. flavus* were subjected to Cluster Amplification Patterns (CAP) analysis for screening of missing regions in the aflatoxin biosynthesis gene cluster [19]. Four multiplex PCRs were designed to amplify 32 markers spaced at 5 kb intervals. The results of the CAP multiplex analysis revealed two atoxigenic strains, Mytoolbox Af01 (from Pivnice, Serbia) and T7/I11, whereas the other isolates were toxigenic (Figure 1). Only one isolate (Mytoolbox Af01) was chosen for biocontrol agent preparation because the two detected atoxigenic strains originated from a narrow geographical region and were genetically identical. The amplification products of the first two multiplex reactions are shown on the gel images, aligned to a schematic diagram of chromosome 3 containing the aflatoxin biosynthesis gene cluster. The CAP analyses showed that the missing cluster in the Mytoolbox Af01 strain and the second atoxigenic strain were over 40 kb. The deletion spans markers AC04 to AC11, which corresponds to the region between genes aflT and verA/aflN (Figure 2).

### 2.2. Quality Control of Atoxigenic Product

Visual evaluations of sporulation observed abundant sporulation on all tested seeds.

### 2.3. Intensity of A. flavus Infection in Maize

*Aspergillus* ear rot infections in the fields were low each year. Statistical analyses showed that there were no significant differences among treatments (at a 95% confidence level).

### 2.4. AFB1 Content

Significant differences in AFB1 content were found between treated and control plots in 2016 and 2017. All 2016 samples from the control plots with and without irrigation contained aflatoxin. A highly significant effect of biocontrol treatment was observed during 2016 and 2017, with an overall reduction of 73%. The reductions achieved in 2016 and 2017 were 83% and 51%, respectively.

In Sombor, the total mycotoxin contamination had mean AFB1 contents of 7.19 ppb and 3.80 ppb in 2016 and 2017, respectively, with no significant differences being found. Similarly, correlation analyses of AFB1 content in 2016 and 2017 were not significant.

We assumed irrigation would help plants avoid infection with the toxigenic *A. flavus*, which should have resulted in lower contamination levels. However, this assumption was incorrect because mean AFB1 content was higher, but not significantly different, in the Sombor treated irrigated plots in 2016 and 2017. However, the AFB1 contents in the unirrigated control plots were slightly, but not significantly, higher. The AFB1 data were logarithmically transformation and analysed using a mixed ANOVA, with 2016 and 2017 considered as repeated measurements. This method enabled analysis of biocontrol and irrigation as main effects.

Table 1 presents t-test results for equality of means and shows that application of the biocontrol agent significantly affected AFB1 levels. Irrigation had no significant effects.

**Figure 3:**
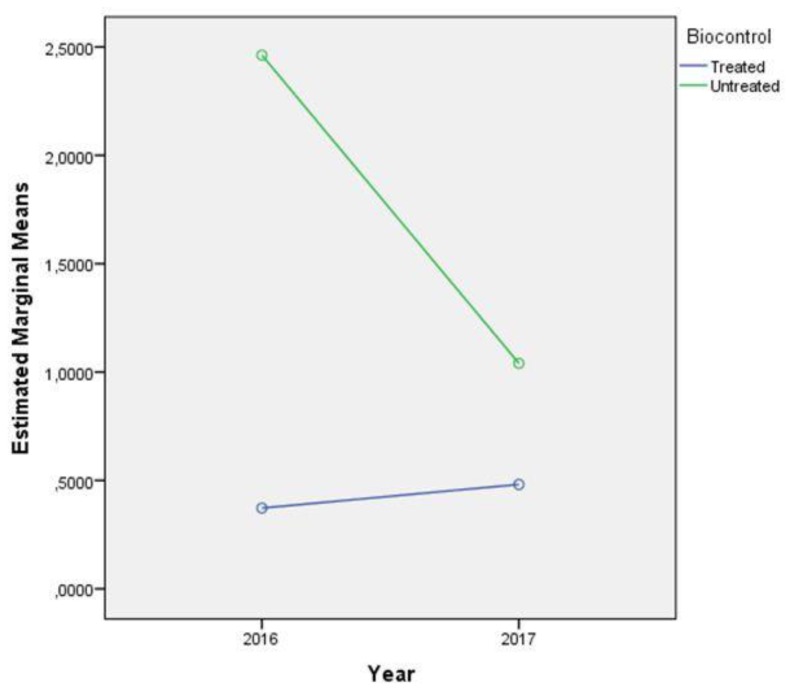
Multivariate test showing the influence of biocontrol on AFB1 contamination levels in 2016 and 2017.

**Figure 4:**
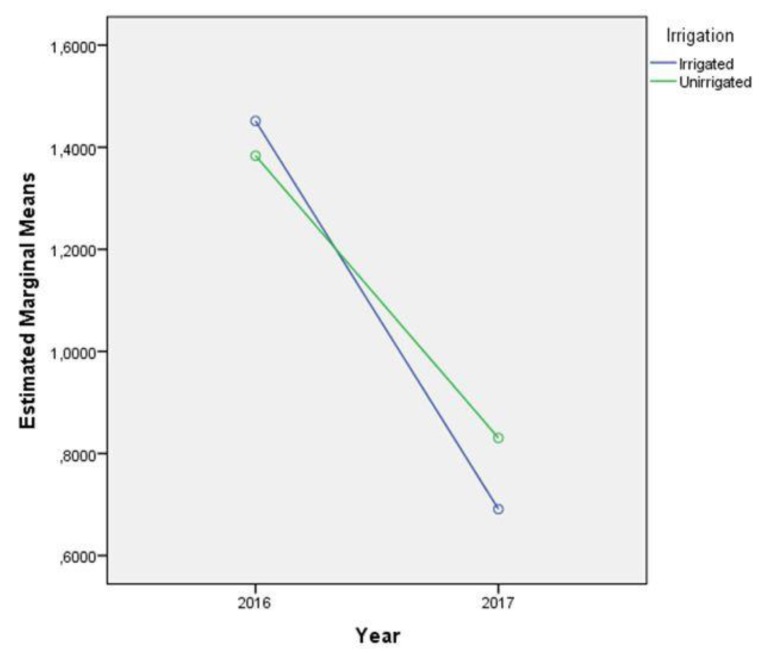
Multivariate test showing the influence of irrigation on AFB1 contamination levels in 2016 and 2017.

Results of the multivariate analyses are shown in Figure 3 and Figure 4. The tests showed that changes in mycotoxin AFB1 contamination with time only had significant interactions with the biocontrol treatment (*p =* 0.04, Wilks’ Lambda = 0.746, df = 1, F = 9.554, η_p_^2^ = 0.254). Contamination levels were also significantly affected by year (*p* = 0.013, Wilks’ Lambda = 0.799, df = 1, F = 7.031, η_p_^2^ = 0.201). The interactions of year and irrigation (*p =* 0.679, Wilks’ Lambda = 0.994, df = 1, F = 0.175, η_p_^2^ =0.006), plus year, biocontrol, and irrigation, were not significantly affected by contamination (*p =* 0.779, Wilks’ Lambda = 0.997, df = 1, F = 0.80, η_p_^2^ = 0.003). Figure 3 shows AFB1 contamination levels in the control plots being heavily depended on the weather conditions during 2016 and 2017, whereas contamination levels in the treated plots remained low and stable in both years.

A between-subject ANOVA (Table 2) for both years showed significant effects in biocontrol application, whereas effects were not significant for irrigation, or biocontrol combined with irrigation.

### 2.5. Climate Conditions

**Figure 5:**
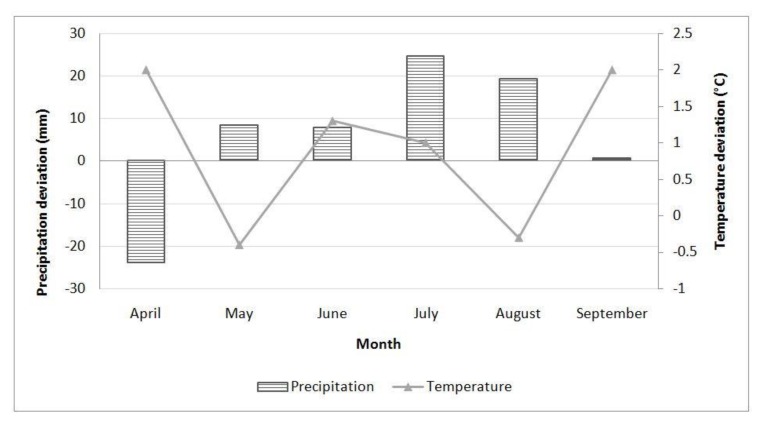
Deviation of total precipitation (columns) and average daily air temperature (lines) from the multiannual average (1981–2010) in Sombor during 2016.

In Sombor during 2016 (Figure 5), precipitation levels in the months during the vegetation period, except for April, were above the multiannual monthly average. Average daily air temperatures were above the long-term average during June and July.

**Figure 6:**
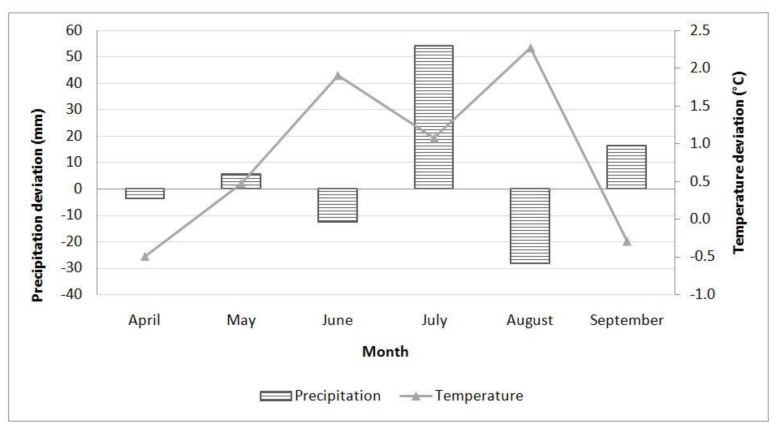
Deviation of total precipitation (columns) and average daily air temperature (lines) from the multiannual average (1981–2010) in Sombor during 2017.

Total rainfall during 2017 in Sombor (Figure 6) was lower than the multiannual average in April, June, and August; however, July rainfall was higher than the average. Average daily air temperatures was the long-term average from May till August.

## 3. Discussion

Mytoolbox Af01 is the first native atoxigenic *A. flavus* strain used as a biocontrol agent to mitigate aflatoxin contamination in Serbian maize fields. The atoxigenic potential of this strain was confirmed by the CAP analyses, which revealed the deletion of 40 kb from the aflatoxin biosynthesis gene cluster. Different types of large deletions in the aflatoxin biosynthesis gene cluster occur often [19,20,21,22], but degeneration of this gene cluster can also be caused by multiple smaller deletions or SNP mutations [23].

The biological control product based on the Mytoolbox Af01 strain was applied in one Serbian location during two consecutive years to assess its aflatoxin reduction potential. *Aspergillus* ear rot was examined every year, but the natural infection rates were low and no significant differences were observed among treatments. AFB1 content in collected samples was determined using a DAS (double antibody sandwich) ELISA test, which was found to be precise in terms of reproducibility. AFB1 was detected in samples from 2016 and 2017, even though there were no visible symptoms of *Aspergillus* ear rot on maize cobs during harvest. Sometimes, seemingly healthy maize grains can contain small levels of AFB1 [24,25]. These results point to the differences between symptomatic and asymptomatic plants and indicates that AFB1 content needs to be investigated further in environmental conditions specific to Serbian maize growing areas. Aflatoxin contamination levels were significantly affected by the application of the bioproduct. Application of the biocontrol product led to an overall mean reduction of 73% in AFB1 levels (83% in 2016 and 51% in 2017). The reduction of aflatoxin contamination from similar biocontrol products has been reported previously. In the USA, Dorner et al. [26] successfully applied an *A. flavus* atoxigenic strain that reduced aflatoxin contamination up to 87%. Moreover, four native atoxigenic strains applied in Nigeria reduced aflatoxin content 67–95% in treated crops [27]. Abbas et al. [28] reported 65–94% reductions of aflatoxin in maize ears inoculated with atoxigenic and toxigenic isolates.

Irrigation in the current experiment did not influence aflatoxin contamination in either year, in contrast to other studies that found irrigation reducing aflatoxin contamination [29,30,31,32]. These contradicting results could have been caused by different methodologies being used, timing and amount of irrigation, plant genotype, weather conditions, soil type, deficiency of easily accessible water in the soil, or other factors. In the control plots, AFB1 contamination levels were dependent on climate conditions during the different years, whereas contamination levels in the treated plots low and stable in both years.

## 4. Conclusions

Mytoolbox Af01 is the first native atoxigenic strain used as a biological preparation to produce significant reductions in aflatoxin levels in a Serbian field. The results indicate that the biocontrol product has a high potential for reducing aflatoxin contamination in local environmental conditions.

## 5. Materials and Methods

### 5.1. Selection of Aspergillus flavus Strains

A selection of *A. flavus* isolates (from maize in Serbia) that were characterised based on colony and spore morphology were selected for this study. Isolates were grown on 5–2 medium [33], made of V-8^TM^ juice (vegetable juice from eight vegetables) containing 2% NaCl and grown for 8–10 days at 31 °C. PCR analyses using species-specific primer Aflafor and universal reverse primer Bt2b [34] were performed to confirm identification. Reactions consisted of: 2 µL of DreamTaq Buffer, 4 µL of dNTP mix, 2 µL of each primer, 5 µL of DNA-free water, 0.2 µL of DreamTaq DNA polymerase and 1 µL DNA template. The samples were subjected to 3 min at 94 °C; 35 cycles of 30 s 94 °C, 30 s 64 °C, 20 s 72 °C; followed by 2 min at 72 °C. The products were visualised on 1% agarose gel in 0.5 x TAE buffer.

### 5.2. Monitoring Deletions in the Aflatoxin Biosynthesis Isolate Mytoolbox Af01 Gene Cluster

Cluster Amplification Patterns (CAP) analyses were performed for screening missing regions in the aflatoxin biosynthesis gene cluster, according to the method by Callicot and Cotty [19]. Four multiplex PCRs were designed to amplify 32 markers. Each 10 µl pre-amplification reaction contained: 0.08 µmol^−1^ of each primer, 1 × AccuStart II PCR SuperMix (Quanta Biosciences, Gaithersburg, MD, USA) and 6 ng genomic DNA. PCR reactions were carried out with the following thermal profile: 94 °C for 1 min, followed by 30 cycles of 94 °C for 30 s, 62 °C for 90 s, 72 °C for 90 s and the final extension step of 72 °C for 10 min. Products were visualised on 1.4% agarose in 1× sodium boric acid buffer [35].

### 5.3. Biocontrol Product Preparation

A biocontrol product with atoxigenic *A. flavus* strain was produced according to the modified method described by Garber et al. [36]. Baked sorghum seeds were used as the inoculum carrier. Prior to inoculation, atoxigenic *A. flavus* strain was cultivated on 5–2 media and incubated at 31 °C for five days. Spore production was performed on sorghum seeds with moisture levels adjusted to 20% using spores from five-day-old cultures in a suspension that was added to autoclaved and cooled sorghum seeds. Flasks were sealed with sterile Tyvek membrane to control humidity levels but allow gas exchange, and incubated for seven days at 35 °C. The spore suspension for biocontrol production was prepared by harvesting spores with 100 mL of sterile 0.5% Tween-80 solution, and a concentration of conidia adjusted to 1–5 × 10^8^ per ml using a haemocytometer. The final suspension was mixed with the sorghum seeds, a seed polymer, and a dye. The moisture content of the final product was adjusted to 10%. The dye was used to indicate the sorghum seeds treated with the atoxigenic *A. flavus*.

### 5.4. Quality Control of the Atoxigenic Biocontrol Product Following Cotty (pers. comm.) 

Prior to application, the quality of the biocontrol product was measured by development and sporulation of Mytoolbox Af01 on sorghum seeds. Individual sorghum seeds of the final biocontrol product were placed in a multi-well plate that had sterile water poured in the outer wells to increase humidity and promote sporulation. Plates were incubated in a closed plastic container at 31 °C for 7 days. Sporulation was visually recorded [37].

### 5.5. Sowing Maize and Application of the Atoxigenic Isolate

The biocontrol product based on atoxigenic *A. flavus* was applied to one maize hybrid (Kerbanis FAO class 500). This hybrid belongs to the FAO group of maize that farmers sow on large areas in Serbia. The maize seeds were planted over two years in one location in Sombor (Serbia). Sowing was completed on 24th April and 11th April in 2016 and 2017, respectively.

When the plants within the inoculated plots had developed 10 leaves, the atoxigenic isolate was manually applied at 10 kg/ha to the soil surface next to each plant. In both years the biocontrol product was applied in the fields with and without irrigation. Irrigation was sufficient to keep the soil at an optimal moisture level for normal plant growth before and after bioproduct application. A drip irrigation system was used between plant rows. The total water volume applied during the vegetation period was 95 l/m^2^ in 2016 and 140 l/m^2^ in 2017. Each of the four combinations irrigated with bioproduct application, irrigated control (without bioproduct application), unirrigated with bioproduct application, and unirrigated control (without bioproduct application) were repeated eight times on individual plots that were over 50 m^2^.

### 5.6. Evaluation of Intensity of A. flavus Infection in Maize

The intensity of *A. flavus* infection on maize cobs was visually evaluated 7–10 days before harvest each year. Disease intensity was evaluated by rating 100 randomly chosen ears within each plot (32 individual plots) using a scale from 1 to 7 [38]. Each ear was evaluated based on the percentage of infected kernels: 1) ear without symptoms, 2) 1–3% infected kernels, 3) 4–10% infected kernels, 4) 11–25% infected kernels, 5) 26–50% infected kernels, 6) 51–75% of infected kernels, 7) 76–100% infected kernels.

### 5.7. Harvest and Samples Preparation

The maize were harvested each year in September. After the harvest, 2 kg samples were taken from 32 individual plots. About 100 g of laboratory samples were prepared by grinding in a laboratory mill with pore diameter of 0.8 mm until >93% of the sample passed through the sieve. The sample was next homogenised by mixing and packed into plastic bags. Samples were stored in a freezer at −20 °C until analysis. Prior to each analysis, the samples were allowed to reach room temperature. Afterwards, the AFB1 content was determined by the ELISA test.

### 5.8. ELISA Test

Exactly 20 g of each ground sample was weighed in a 150 mL beaker. Aflatoxin B1 was extracted with 100 mL of 70% methanol solution on an Ultra Turrax T18 homogeniser (IKA, Staufen, Germany) for 3 min at 11,000 rpm. The crude extract was filtered through a quantitative slow filtration filter paper (Filtros Anoia, Barcelona, Spain).

The immunochemical analysis was performed using the AgraQuant® Aflatoxin B1, Quantitative Test Kit (Romer Labs, Tulln, Austria) with four calibration standard solutions (0, 2, 5, 20, and 50 ppb). The analytical procedure was carried out according to the manufacturer’s instructions. Aflatoxin quantification was done on an ELISA reader equipped with a 450 nm filter (BioTec Instruments, USA). To ensure the quality of the results, the aflatoxin was validated in the laboratory. Validation parameters were evaluated according to the European Commission [39]. The limit of quantification (LOQ) of 2 µg/kg that was established by the manufacturer, was experimentally verified by analysing blank samples of corn that were treated with 2 µg/kg of an aflatoxin B1 standard solution (Sigma Aldrich, St. Louis, MO, USA). Samples containing less than 2 µg/kg aflatoxin were considered negative. The average trueness of this method was calculated by analysing eight successive corn samples from a certified reference material (CRM) coded TR-A100 (Trilogy lab, Washington, MO, USA). The average trueness of 105.4% was within acceptable limits according to the European Regulation [39]. In-house reproducibility was assessed after eight successive CRM tests, on two different occasions, resulting in 16 tests. The obtained HORRAT_R_ value of 0.20 was within the acceptable criteria for reproducibility established by the European Regulation [39].

### 5.9. Statistical Analyses

Statistical analyses of the scoring data from the field maize infection and AFB1 content were performed in Statistica v. 13 (TIBCO Software Inc., Silicon Valley, CA, USA, 2017). Because the maize infection data were non-parametric, Kruskal–Wallis tests were used to determine if the mean ranks of the infection levels were the same in all treatments. Furthermore, multiple comparisons of mean ranks were used as post hoc tests to determine the treatments that were significantly different from each other. The aflatoxin content data were logarithm transformation and used in an analysis of variance to determine differences between treatments, and a multivariate test to assess between-subject effects.

### 5.10. Climate Conditions

Aflatoxin contamination in maize is correlated with plant, drought, and heat stress [40]. Therefore temperature and precipitation were monitored during the vegetation growth period at the locality every year. Data were obtained from a Metos® automatic weather station (Metos®, Pessl Instruments, Weiz, Austria) and compared to multiannual averages for 1981–2010 [41].

## Figures and Tables

**Table 1:** T-test for equality of means of different AFB1 contamination levels.

Effect	N	Mean	Standard Deviation	*t*-Test for Equality of Means		
				t	df	Significance Probability (2-Tailed)
**Biocontrol**						
Treated	32	2.31	6.713	−3.858	62	0.000
Untreated	32	8.68	6.483			
**Irrigation**						
Irrigated	32	5.75	8.215	0.279	62	0.782
Unirrigated	32	5.24	6.355			

**Table 2:** Between-subject ANOVA test of the influence of different factors on AFB1 contamination levels.

Effect	Type III Sum of Squares	df	Mean Square	F	Significance Probability	Partial Eta Squared
Intercept	75.913	1	75.913	98.064	0.000	0.778
Biocontrol	28.057	1	28.057	36.244	0.000	0.564
Irrigation	0.020	1	0.020	0.026	0.873	0.001
Biocontrol*Irrigation	0.342	1	0.342	0.442	0.512	0.016
Error	21.675	28	0.774			
